# Nomograms for Predicting Overall Survival and Cancer-Specific Survival of Patients With Renal Cell Carcinoma and Venous Tumor Thrombus: A Population-Based Study

**DOI:** 10.3389/fsurg.2022.929885

**Published:** 2022-06-08

**Authors:** Lin Yang, Bin Fu

**Affiliations:** Department of Urology, The First Affiliated Hospital of Nanchang University, Nanchang, China

**Keywords:** renal cell carcinoma, venous tumor thrombus, cancer-specific survival, overall survival, nomogram

## Abstract

**Background:**

To provide better prognostic information for patients with renal cell carcinoma (RCC) combined with venous tumor thrombus (VTT). In turn, guide patients’ families and doctors to formulate plans for follow-up treatment and follow-up. We developed nomograms to predict cancer-specific survival (CSS) and overall survival (OS).

**Methods:**

A total of 2961 cases were included in this study. Through univariate and multivariate Cox proportional hazard regression analysis, independent risk factors affecting CSS and OS were screened out, and then a nomogram was drawn based on the screened variables.

**Results:**

Independent risk factors affecting CSS include: tumor size (HR = 1.05), histology (HR = 1.75), grade (HR = 1.94), N staging (HR = 2.06), and M staging (HR = 2.87). The median survival time for CSS was 106 months. Independent risk factors for OS include age (HR = 1.60), tumor size (HR = 1.04), histology (HR = 1.60), grade (HR = 1.68), N staging (HR-1.99), M staging (HR = 2.45). The median survival time for OS is 67 months.

**Conclusions:**

The nomogram based on independent risk factors affecting CSS and OS can well predict the prognosis of renal cell carcinoma with venous tumor thrombus.

## Background

Globally, renal cell carcinoma is the 9th most common cancer in men and the 14th most common cancer in women worldwide (1). Approximately 4%–10% of cases were diagnosed with venous tumor thrombus at initial diagnosis (2), and nearly one-third of these patients have concurrent metastatic disease. Although venous system involvement is a late manifestation of the disease, radical nephrectomy with tumor thrombus removal remains the standard treatment for disease control (3).

A meta-analysis pointed out that the 5-year cancer-specific survival (CSS) after surgery was only 25%–53% for these cases (4), which is not optimistic compared with the postoperative survival rate of localized renal cell carcinoma. To evaluate the prognosis of patients with RCC with VTT, E. Jason Abe (5) developed a Nomogram diagnostic chart for predicting postoperative recurrence of non-metastatic RCC with tumor thrombus, and Ahmed Q. Haddad (6) developed a preoperative multivariable prognostic model for prediction of survival and major complications following surgical resection of RCC with VTT. Most of these studies were from single-center or small cohorts, and there was heterogeneity among the prognostic risk factors proposed by different studies.

To help patients and physicians decide on adjuvant therapy, the type and frequency of follow-up, and to provide patients and their families with useful information about treatment modalities and short-term and long-term outcomes, we developed a nomogram predicting tumor-specific and overall survival based on the SEER database.

## Methods

### Patient Population and Data Collection

The SEER database from National Cancer Institute is the most authoritative source of information about cancer incidence and survival in the United States (https://seer.cancer.gov/), and the SEER database is a public database, so no ethical review is required. We logged in to the SEER*stat 8.4.0 software through the account (22230-Nov2020) obtained by registration to extract the required tumor data. Inclusion criteria for the data: (1) over 18 years old, (2) the primary site of the tumor in the kidney, (3) with venous tumor thrombus, (4) having undergone radical nephrectomy (including minimally invasive and open surgery), (5) cases were diagnosed between 2005 and 2015. Exclusion criteria for data: (1) missing important data, (2) bilateral tumors, (3) postoperative pathology suggests tumors of non-renal origin. According to the inclusion and exclusion criteria, a total of 2,961 eligible cases were screened in this study.

### Variables

We obtained the following case information from the SEER database: age, race, sex, year of diagnosis, Laterality, CS tumor size (2004–2015), CS extension (2004–2015), histology, grade, N staging (AJCC, 6th ed.), M staging (AJCC, 6th ed.), cause-specific death, vital status, survival months. Because renal cell carcinoma patients over 65 years old have significantly higher Microvascular density than younger (<65 years old) patients. Microvascular density is an important factor affecting the occurrence and development of tumors, so the age of 65 is a key node (7). Age was divided into two groups (≤65 years vs >65 years), the race was divided into white group and other groups, sex included male and female, and year of diagnosis was divided into two groups (2005–2010 vs 2011–2015), tumor Laterality includes left and right. Tumor size was converted to centimeters according to the code, venous tumor thrombus was divided into two groups (under the diaphragm vs above the diaphragm) according to the code (600, 601, 610, 640, 620, 645), histology was divided into clear cell carcinoma group and other groups, tumor nuclear grades include grade I, grade II, grade III, and grade IV, and were divided into two groups (I/II vs III/IV). Tumor without lymph node metastasis was defined as N0, lymph node metastasis was defined as N1, no distant metastasis was defined as M0, and distant metastasis was defined as M1. Cause-specific death includes alive (or death of other cause) and dead (attributable to this cancer), the latter being defined as the primary outcome of cancer-specific survival (CSS). Vital status includes alive and dead, the latter being defined as the primary outcome of overall survival (OS). Survival months refer to how long a patient survives during the follow-up period.

### Statistical Analysis

Continuous variables were presented as means ± standard deviations and categorical variables as frequency (percentage). Clinical variables associated with CSS and OS were assessed based on clinical importance, scientific knowledge, and predictors identified in previously published articles (8–10). Univariable and multivariable Cox proportional hazards regression model analyses were performed for CSS and OS. Univariate predictive variables with *p* < 0.05 were applied to multivariate analyses to identify the independent prognostic factors. Hazard ratios (HRs) were presented with their 95% CIs. Then, a nomogram model was established using the independent risk factors screened out by the multivariable Cox proportional hazards regression model (rms in R, version 4.1.3; http://www.r-project.org). In addition, computing the C statistic estimates the probability that the predicted outcome agrees with the observed outcome. Bootstrap validation (1,000 resamples) was performed and nomogram performance was determined using a calibration plot of observed versus predicted probabilities. Finally, we drew Kaplan-Meier curves over the tertiles of patients stratified according to the nomogram prediction scores in the dataset to further assess calibration, and differences were examined using the log-rank test. Statistical analyses were performed with software programs (R, version 4.1.3; http://www.r-project.org). All tests were 2-sided, and *p* < 0.05 was considered statistically significant.

## Results

In this study, a total of 2,961 patients with renal cell carcinoma with venous tumor thrombus were included. The clinicopathological characteristics of our study cohort are shown in Table 1. 44.3% of the cases in the cohort were older (>65 years) and mostly white (82.9%). Males (68.9%) accounted for the majority of these patients. The average size of the tumors was 8.78 cm (±3.53 cm), and about half of them were located on the left side (50.7%). The tumor tissues were dominated by clear cells (92.1%), and most of them belonged to grade III/IV (70.7%). Among these cases, 13.7% had lymph node metastasis (N1), 22.7% had distant metastasis (M1), and 1% had an adrenal invasion. The venous tumor thrombus of most tumors was located below the diaphragm (96.5%). At the end of follow-up, 43.4% of patients died from renal cell carcinoma and 56.5% from all causes, with a mean survival time of 56.7 months (±42.05 months). The CSS ratios at 1, 3 and 5 years were 85.6%, 70.1% and 60.2%. The OS rates at 1, 3, and 5 years were 83.3%, 65.4%, and 52.8%. Median survival for CSS was 106 months (95% CI, 91–123 months) and median survival for OS was 67 months (95%, 62–73 months).

**Table 1:** Clinicopathological features of renal cell carcinoma patients with venous tumor thrombus.

Variables	Value (*n* = 2,961)
Age (years)	
≤65	1,650 (55.7%)
>65	1,311 (44.3%)
Race	
White	2,455 (82.9%)
Others	506 (17.1%)
Sex	
Female	922 (31.1%)
Male	2,039 (68.9%)
Laterality	
Left	1,502 (50.7%)
Right	1,459 (49.3%)
Tumor size(cm)	8.78 ± 3.53
Histology	
Clear cell adenocarcinoma	2,728 (92.1%)
Others	233 (7.9%)
Grade	
I/II	868 (29.3%)
III/IV	2,093 (70.7%)
N stage	
N0	2,555 (86.3%)
N1	406 (13.7%)
M stage	
M0	2,290 (77.3%)
M1	671 (22.7%)
Violation of adrenal glands	
NO	2,930 (99.0%)
YES	31 (1.0%)
Venous tumor thrombus	
Below the diaphragm	2,857 (96.5%)
Above the diaphragm	104 (3.5%)
Cancer-specific survival	
Alive	1,677 (56.6%)
Dead	1,284 (43.4%)
Overall survival	
Alive	1,288 (43.5%)
Dead	1,673 (56.5%)
Survival months	56.7 ± 42.05

*Continuous variables were presented as means ± standard deviations and categorical variables as frequency (percentage)*.

Shown in Tables 2, 3 are cox proportional hazards regression models for variables versus CSS,OS. First, univariate regression analysis was performed to screen out variables with *p* < 0.05, and then they were included in multivariate regression analysis. Finally, the following independent risk factors for CSS were obtained: tumor size (HR: 1.05, 95% CI, 1.04 –1.07, *p* < 0.001), histology (HR:1.75, 95%CI, 1.47–2.08, *p* < 0.001), grade (HR:1.94, 95% CI, 1.67–2.26, *p* < 0.001), N_stage (HR:2.06, 95% CI, 1.79–2.36, *p* < 0.001), M_stage (HR:2.87, 95% CI, 2.54–3.25, *p* < 0.001). The following were independent risk factors for OS: age (HR: 1.60, 95%CI, 1.45–1.77, *p* < 0.001), tumor size (HR:1.04, 95%CI, 1.03–1.05, *p* < 0.001), histology (HR:1.60, 95%CI, 1.37 –1.88, *p* < 0.001), grade (HR:1.68, 95%CI, 1.48–1.89, *p* < 0.001), N_stage (HR:1.99, 95% CI, 1.74–2.26, *p* < 0.001), M_stage (HR:2.45, 95% CI, 2.19–2.74, *p* < 0.001).

**Table 2:** Cox proportional hazards regression model showing the association of variables with cancer-specific survival.

Variable	Univariable				Multivariable			
	HR	95% CI		*p*-value	HR	95% CI		*p*-value
Factors Selected								
Tumor size (cm)	1.11	1.09	1.12	<0.001	1.05	1.04	1.07	<0.001
Histology								
Clear cell adenocarcinoma	1 [Reference]				1 [Reference]			
Others	2.41	2.03	2.85	<0.001	1.75	1.47	2.08	<0.001
Grade								
I/II	1 [Reference]				1 [Reference]			
III/IV	2.80	2.42	3.23	<0.001	1.94	1.67	2.26	<0.001
N stage								
N0	1 [Reference]				1 [Reference]			
N1	3.99	3.51	4.43	<0.001	2.06	1.79	2.36	<0.001
M stage								
M0	1 [Reference]				1 [Reference]			
M1	4.19	3.74	4.69	<0.001	2.87	2.54	3.25	<0.001
Factors Not Selected								
Age								
≤65 years	1 [Reference]							
>65 years	1.07	0.96	1.19	0.24				
Race								
White	1 [Reference]							
Others	1.09	0.95	1.26	0.23				
Sex								
Female	1 [Reference]							
Male	0.99	0.88	1.11	0.81				
Laterality								
Left	1 [Reference]							
Right	0.90	0.81	1.01	0.07				
Violation of adrenal glands								
N0	1 [Reference]				1 [Reference]			
YES	3.20	2.15	4.75	<0.001	0.97	0.65	1.45	0.88
Venous tumor thrombus								
Under the diaphragm	1 [Reference]				1 [Reference]			
Above the diaphragm	1.62	1.25	2.11	<0.001	1.17	0.89	1.53	0.26

*HR, hazard ratio; CI, confidence interval*.

**Table 3:** Cox proportional hazards regression model showing the association of variables with overall survival.

Variable	Univariable				Multivariable			
	HR	95% CI		*p*-value	HR	95% CI		*p*-value
Factors Selected								
Age								
≤65 years	1 [Reference]				1 [Reference]			
>65 years	1.35	1.23	1.49	<0.001	1.60	1.45	1.77	<0.001
Tumor size (cm)	1.07	1.06	1.09	<0.001	1.04	1.03	1.05	<0.001
Histology								
Clear cell adenocarcinoma	1 [Reference]				1 [Reference]			
Others	2.16	1.85	2.16	<0.001	1.60	1.37	1.88	<0.001
Grade								
I/II	1 [Reference]				1 [Reference]			
III/IV	2.15	1.91	2.42	<0.001	1.68	1.48	1.89	<0.001
N stage								
N0	1 [Reference]				1 [Reference]			
N1	3.23	2.86	3.64	<0.001	1.99	1.74	2.26	<0.001
M stage								
M0	1 [Reference]				1 [Reference]			
M1	3.22	2.90	3.57	<0.001	2.45	2.19	2.74	<0.001
Factors Not Selected								
Race								
White	1 [Reference]							
Others	1.11	0.98	1.26	0.10				
Sex								
Female	1 [Reference]							
Male	0.97	0.87	1.07	0.50				
Laterality								
Left	1 [Reference]							
Right	0.96	0.88	1.06	0.45				
Violation of adrenal glands								
N0	1 [Reference]				1 [Reference]			
YES	2.53	1.71	3.76	<0.001	0.83	0.55	1.24	0.35
Venous tumor thrombus								
Under the diaphragm	1 [Reference]				1 [Reference]			
Above the diaphragm	1.58	1.25	2.00	<0.001	1.23	0.97	1.56	0.09

*HR, hazard ratio; CI, confidence interval*.

**Figure 1:**
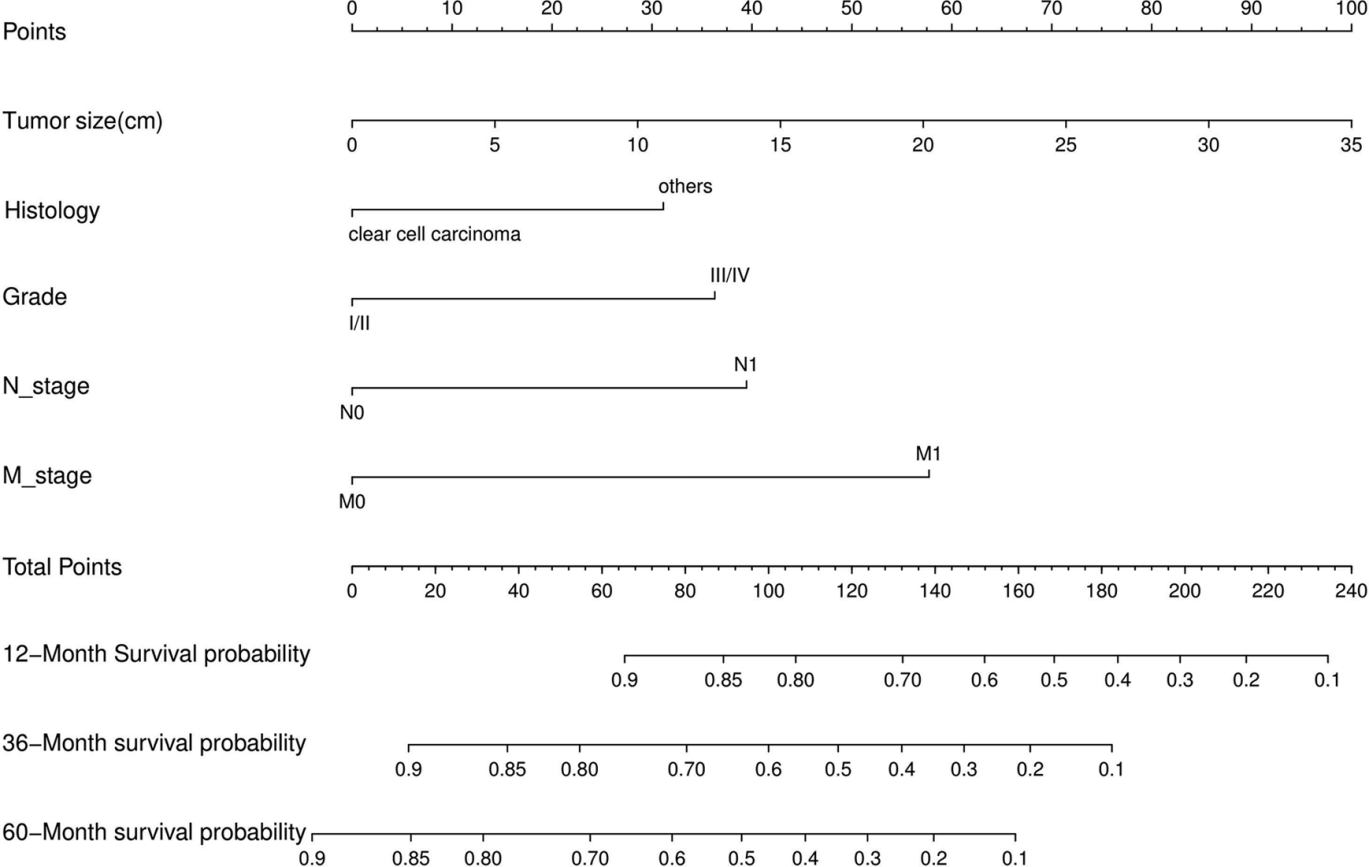
Nomogram for predicting postoperative CSS in patients with renal cell carcinoma complicated with venous tumor thrombus.

**Figure 2:**
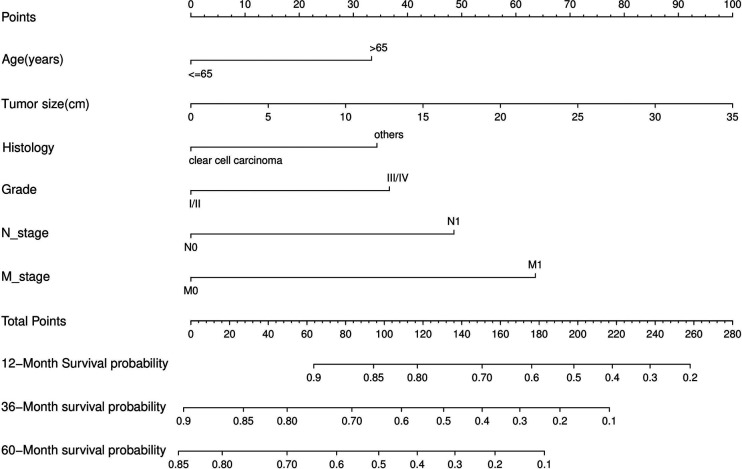
Nomogram for predicting postoperative OS in patients with renal cell carcinoma complicated with venous tumor thrombus.

**Figure 3:**
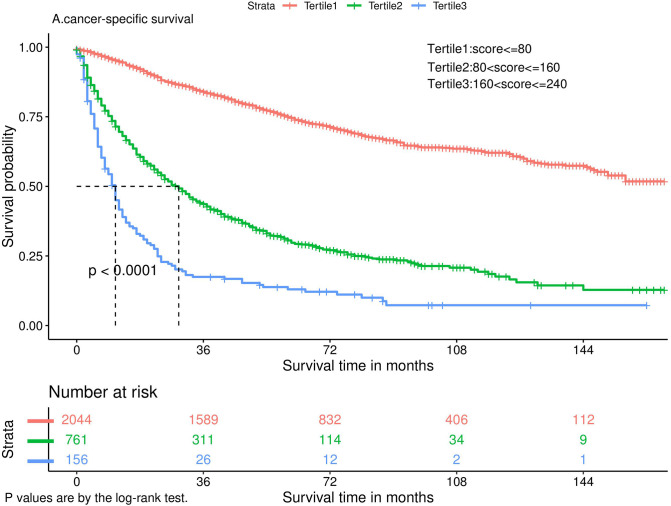
Kaplan-Meier curve.

**Figure 4:**
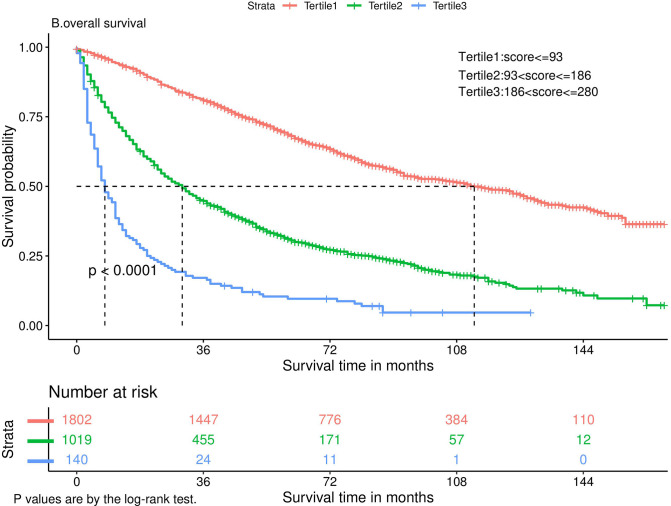
Kaplan-Meier curve.

**Figure 5:**
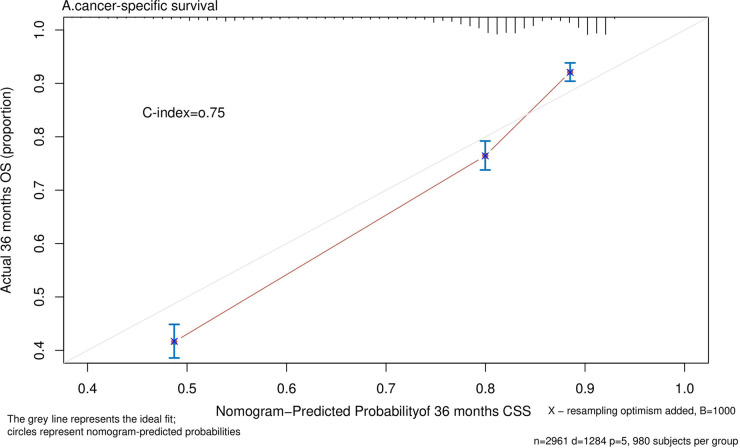
Calibration plot comparing predicted and actual survival probability at 36 months follow-up.

**Figure 6:**
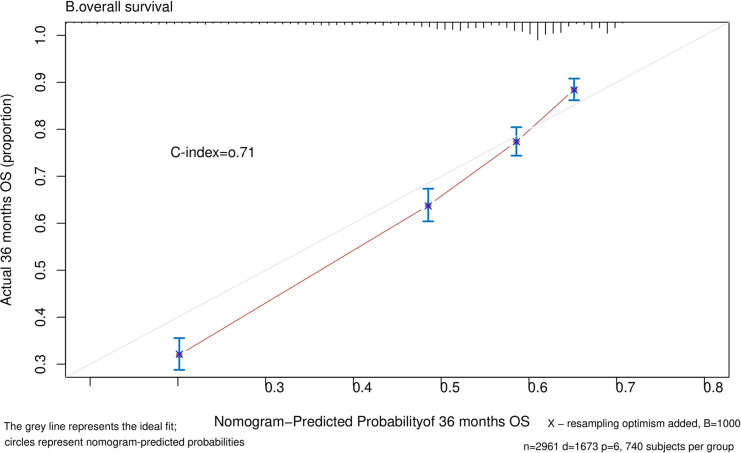
Calibration plot comparing predicted and actual survival probability at 36 months follow-up.

Nomograms predicting CSS and OS at 1, 3, and 5 years after surgery in patients with renal cell carcinoma with venous tumor thrombus were shown in Figures 1, 2. The nomogram for predicting CSS was based on several variables: tumor size, histology (clear cell carcinoma or other), grade (I/II or III/IV), N_stage (N0 or N1), M_stage (M0 or M1). Likewise, nomograms for predicting OS were based on several variables: age (≤65 or >65 years), tumor size, histology (clear cell carcinoma or other), grade (I/II or III/IV), N_stage (N0 or N1). As assessed by the nomogram, the higher the cumulative score, the higher the risk of poor prognosis. To verify the predictive accuracy of the nomogram, we calculated each patient’s score and then plotted Kaplan-Meier curves against the tertiles of the total score, as shown in Figures 3, 4. In the CSS curve, the median survival time did not appear for tertile 1, the median survival time for tertile 2 was 29 months, and the median survival time for tertile 3 was 11 months. In the OS curves, median survival was 113 months for tertile 1, 30 months for tertile 2, and 8 months for tertile 3. Both log-rank test *p*-values were less than 0.0001. In addition, the discriminative ability of the model for CSS and OS was also evaluated with the C statistic (CSS = 0.75, OS = 0.71). Finally, bootstrap was performed by 1,000 resampling validation, evaluating the accuracy of the model. The bootstrapped calibration plots for predicting 3-year CSS and OS were shown in Figures 5, 6. To compare the changes in CSS and OS of patients between 2005–2010 and 2011–2015, Figures 7, 8 showed the survival curves of the two. Median survival was similar between the two groups in the CSS curve (93 vs 97 months, *p* = 0.24), as was the OS curve (70 vs 66 months, *p* = 0.26).

**Figure 7:**
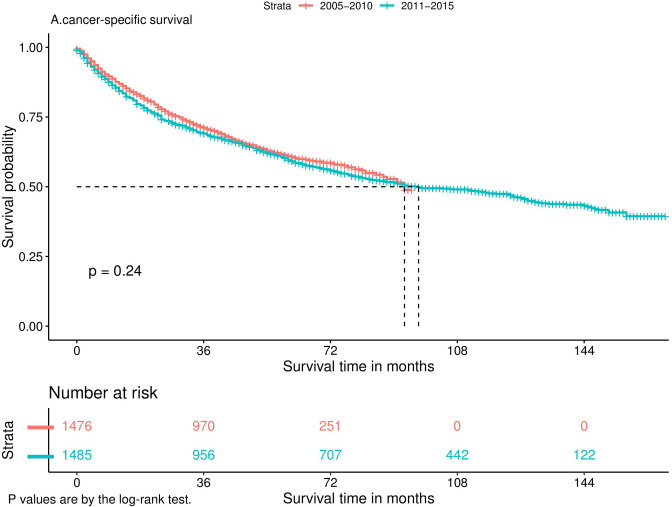
Kaplan-Meier curve.

**Figure 8:**
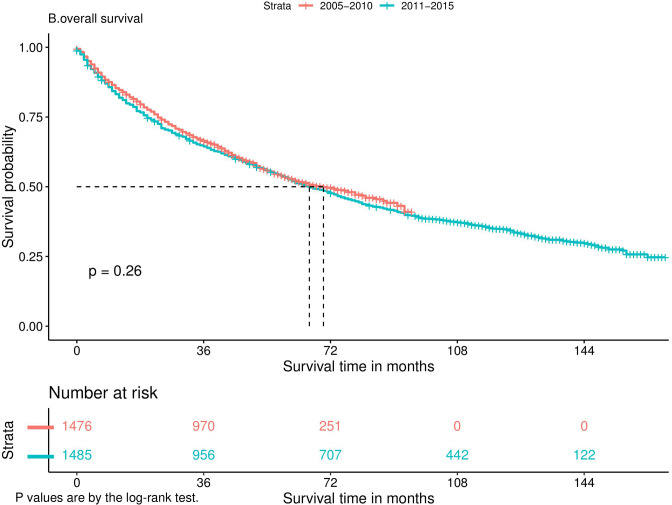
Kaplan-Meier curve.

## Discussion

With the advancement of surgical techniques and instruments, radical nephrectomy combined with tumor thrombus removal has become the standard treatment for patients with renal cell carcinoma combined with venous tumor thrombus (11). Although these patients have achieved a significant survival benefit relative to patients who have not undergone surgery (12), overall, the long-term survival rate of patients with tumor thrombus is still low (13). In recent years, the rapid development of targeted therapy and immunotherapy has brought new hope for some patients. Some studies have reported that some patients with advanced renal cancer or tumor thrombus can benefit from these two treatments (14, 15). Therefore, conducting postoperative risk stratification for patients with RCC is complicated by VTT to guide follow-up and treatment. We screened 2,961 patients based on the SEER database. After univariate and multivariate Cox proportional hazards regression analysis, we obtained five independent risk factors affecting CSS: tumor size, histology, grade, N_stage, M_stage; six affecting OS Independent risk factors: age, tumor size, histology, grade, N_stage, M_stage, and nomograms of CSS and OS were drawn.

Similar results have been reported in other studies, but the risk factors identified by different studies are quite different, and even the conclusions are opposite. According to the seventh edition of the AJCC Cancer Staging Manual (16), tumor size is an important basis for T1 and T2 staging. Brett et al. (17) reported that almost all tumors >7 cm in diameter and a substantial proportion of tumors <7 cm in diameter showed extrarenal spread. This suggests that the larger the tumor diameter, the higher the degree of malignancy. Weixing et al. (18) also noted that smaller tumors had a better prognosis for patients with metastatic renal cell carcinoma who underwent cytoreductive nephrectomy. The same is true of our findings that tumor size is a key factor affecting CSS and OS in patients. Because larger tumors may mean more aggressiveness, this can significantly increase the risk of recurrence and metastasis after surgery, leading to a worse prognosis.

Regarding the effect of histological subtypes on the prognosis of patients with renal cell carcinoma, although most studies agree that the prognosis of clear cell carcinoma is better, there is also heterogeneity between the results of different studies. According to a study by Dharam et al. (19), patients with non-clear cell carcinoma have larger tumor size, higher nuclear grade, more frequent sarcoid differentiation, and more frequent lymph node invasion, which leads to a worse prognosis. Similarly, studies by the International Renal Cell Carcinoma-venous Thrombus Consortium (8) and Liangyou et al. (20) also suggested that the histological type of non-clear cell carcinoma is an independent risk factor for poor prognosis. However, for patients with tumor thrombosis, the study by Nocera et al. (21) came to different conclusions. The three histologic subtypes, clear cell, papillary, and chromophobe, almost all exhibited poor cancer-specific mortality (CSM) in patients with T3b-T4 RCC, with no clinically meaningful between-group differences in CSM. Our findings suggest that histological type is an independent risk factor for CSS and OS in patients with tumor thrombus, perhaps because Luigi Nocera’s subgroup did not include patients with stage T3a and M1, resulting in heterogeneity of results, so we still tend to think that histological type is a key factor affecting the prognosis of patients with tumor thrombus. In addition, our study found that in patients with renal cell carcinoma and venous tumor thrombus, most tumors had higher nuclear grades (grade = III/IV: 2,093 (70.7%)). Nuclear grade in renal cell carcinoma has been recognized as a prognostic factor for nearly 100 years (22), and our findings also suggest that nuclear grade is an independent risk factor for CSS and OS in patients. From this, it may be reasonable to infer that a higher nuclear grade may be one of the important reasons for the high invasiveness of venous tumor thrombus and the poor prognosis of patients with tumor thrombus.

It is well known that lymph node metastasis in cancer patients often indicates a more aggressive tumor and a worse prognosis. The same is true of our findings. Patients with venous tumor thrombus with lymph node metastasis have a 2.06-fold increased risk of tumor-specific death (HR = 2.06, *p* < 0.001) and a 1.99-fold increased overall mortality risk (HR = 1.99, *p* < 0.001). Arnav’s (23) study of patients with pathological node-positive renal cell carcinoma found that patients with node-negative stage III had better survival (5-year survival: 61.9%), but node-positive stage III (5-year survival: 22.7%) Survival was similar to that of stage IV (5-year survival: 15.6%) RCC patients. They believe that such patients should be further stratified to guide lymph node dissection and subsequent treatment. However, lymph node dissection is not routinely performed during radical nephrectomy because renal tumors metastasize primarily through the hematologic system, and lymph node dissection does not appear to confer any survival benefit for patients (24). On the other hand, a study by the International Renal Cell Carcinoma Venous Thrombus Consortium (25) suggests that lymph node dissection can improve the survival rate of patients with positive lymph nodes. In conclusion, whether lymph node dissection can bring survival benefits to patients with renal cell carcinoma complicated with venous tumor thrombus still needs higher-quality prospective randomized controlled studies to verify.

Generally speaking, when the tumor progresses to the distant metastatic stage, it means that the disease has reached the terminal stage, and the treatment options at this time are limited. In our study, the presence of distant metastases significantly increased tumor-specific mortality (HR = 2.87) and overall mortality (HR = 2.45). Although studies (26) have shown that Cytoreductive Nephrectomy can improve the OS of patients with renal cell carcinoma with distant metastasis and venous tumor thrombus, the survival rate of these patients is still low. However, with the development of targeted therapy and immunotherapy, new treatment options have been brought to patients with advanced cancer. The finding of a recent randomized controlled trial (27) was surprising in that in patients with metastatic renal cell carcinoma, sunitinib alone was not inferior to sunitinib after nephrectomy. This means that some patients with distant metastases can avoid the risks of surgery and improve their quality of life. So, can patients with renal cell carcinoma combined with venous tumor thrombus benefit from it? We plotted survival curves for 2005–2010 and 2011–2015 and found no improvement in CSS (*p* = 0.24) and OS (*p* = 0.26) during these two periods. In other words, the development of targeted drugs did not bring significant survival benefits to patients with tumor thrombi. As shown in Liangyou’s findings (28), postoperative adjuvant sorafenib or sunitinib did not have a survival advantage in patients with non-metastatic renal cell carcinoma and VTT compared with controls. Perhaps, immunotherapy can bring some improvement for these patients. The results of Yoshida’s (29) study showed that patients with RCC and VTT who received immunotherapy all had their tumors shrunk, significantly reducing the risk of surgery. However, whether immunotherapy can improve the long-term survival of such patients still requires prospective studies with larger sample sizes to verify.

Clearly, the higher the level of venous tumor thrombus, the significantly increased perioperative risk (11). However, the effect of the level of venous tumor thrombus on the long-term survival of patients with renal cell carcinoma combined with venous tumor thrombus has been controversial. The International Renal Cell Carcinoma-Venous Thrombosis Consortium (8) believes that the level of tumor thrombus is an independent predictor of survival, and even the T3 stage in the 7th edition of the AJCC Cancer Staging Manual (16) is based on the level of tumor thrombus. However, our study and those from other centers (9, 10) came to a different conclusion that the level of venous tumor thrombus did not significantly affect the CSS and OS of patients. Perhaps the heterogeneity of the results stems from the differences in the classification of tumor thrombus levels in different studies. To better evaluate the prognosis of these patients, a more detailed classification of the T3 stage should be carried out.

There are some limitations to this study. First, this study is a retrospective study based on the SEER database. In the process of data screening, cases with missing important data were excluded, and there may be errors in the variable coding of the database. These factors may lead to the study of the results deviating from the actual situation. Second, the database did not contain detailed information on patients’ continued adjuvant therapy after surgery, a factor that influenced the findings.

## Conclusion

To sum up, for patients with renal cell carcinoma combined with venous tumor thrombus, tumor size, histology, grade, N_stage, and M_stage are independent risk factors for CSS; age, tumor size, histology, grade, N_stage, and M_stage are the independent risk factors for CSS. Independent risk factors for OS. A nomogram based on these factors can be a good predictor of patient prognosis to guide follow-up and treatment.

## Data Availability

The datasets presented in this study can be found in online repositories. The names of the repository/repositories and accession number(s) can be found below: https://seer.cancer.gov/data/.
